# Aerial photography based census of Adélie Penguin and its application in CH_4_ and N_2_O budget estimation in Victoria Land, Antarctic

**DOI:** 10.1038/s41598-017-13380-6

**Published:** 2017-10-11

**Authors:** Hong He, Xiao Cheng, Xianglan Li, Renbin Zhu, Fengming Hui, Wenhui Wu, Tiancheng Zhao, Jing Kang, Jianwu Tang

**Affiliations:** 1 State Key Laboratory of Remote Sensing Science, and College of Global Change and Earth System Science, Beijing Normal University, Beijing, 100875 P. R. China; 2 Joint Center for Global Change and China Green Development, Beijing Normal University, Beijing, 100875 P. R. China; 3 Institute of Polar Environment, School of Earth and Space Sciences, University of Science and Technology of China, Hefei, 230026 P. R. China; 4 Polar surveying and mapping engineering center, Heilongjiang Administration of Surveying, Mapping and Geoinformation, Harbin, 150081 P. R. China; 5 The Ecosystems Center, Marine Biological Laboratory, Woods Hole, MA 02543 USA

## Abstract

Penguin guano provides favorable conditions for production and emission of greenhouse gases (GHGs). Many studies have been conducted to determine the GHG fluxes from penguin colonies, however, at regional scale, there is still no accurate estimation of total GHG emissions. We used object-based image analysis (OBIA) method to estimate the Adélie penguin (*Pygoscelis adeliae*) population based on aerial photography data. A model was developed to estimate total GHG emission potential from Adélie penguin colonies during breeding seasons in 1983 and 2012, respectively. Results indicated that OBIA method was effective for extracting penguin information from aerial photographs. There were 17,120 and 21,183 Adélie penguin breeding pairs on Inexpressible Island in 1983 and 2012, respectively, with overall accuracy of the estimation of 76.8%. The main reasons for the increase in Adélie penguin populations were attributed to increase in temperature, sea ice and phytoplankton. The average estimated CH_4_ and N_2_O emissions tended to be increasing during the period from 1983 to 2012 and CH_4_ was the main GHG emitted from penguin colonies. Total global warming potential (GWP) of CH_4_ and N_2_O emissions was 5303 kg CO_2_-eq in 1983 and 6561 kg CO_2_-eq in 2012, respectively.

## Introduction

Adélie penguin (*Pygoscelis adeliae*) is considered to be an Antarctic icon, a sensitive species to environmental changes in the Southern Ocean^1,2^. It is a circumpolar meso-predator exposed to the full range of the Antarctic climate and is undergoing dramatic population shifts coincident with climate change^3^. Significant changes in Adélie penguin numbers due to climate change could warn of changes in the abundance of their prey and/or the structure and function of the marine ecosystem due to climate factors^4^. Sea animals such as penguins play an important role in the nutrient cycling of ecosystems by transferring carbon (C) and nitrogen (N) from the marine to the terrestrial environment^5–7^. The deposition of large amounts of penguin guano strongly influences the physical and chemical properties of soils in the Antarctic, and every summer the large volume of penguin guano creates favorable conditions for the production and emission of greenhouse gases such as methane (CH_4_) and nitrous oxide (N_2_O) in the soil^5,8,9^. Therefore, marine animal colonies are hot spots for CH_4_ and N_2_O emissions in maritime Antarctica, or even at the global scale, and current climate warming will further increase their emissions^10^.

Field observations have indicated that penguin colonies were significant sources of atmospheric CH_4_ and N_2_O compared to other areas, such as tundra without penguins^8,11–15^. For example, Zhu *et al*.^5^ measured net CH_4_ fluxes from penguin colonies during the summertime of 2005/2006 using a static chamber technique, and found that the CH_4_ emission rates from these colonies were much higher than from the normal tundra without penguin colonies. Laboratory incubation experiments have further confirmed that the soils beneath penguin colonies produced much higher N_2_O and CH_4_ emissions than tundra soils without penguin colonies^10^. The mean CH_4_ fluxes were 0.4–2.4 mg CH_4_-C m^−2^ h^−1^ for penguin guano and 2.4–17.7 μg CH_4_-C m^−2^ h^−1^ for the colony soils, i.e., the fluxes from penguin guano were two orders of magnitude higher than those from the colony soils^16^. Meanwhile, Zhu *et al*.^14^ made a rough estimate of potential greenhouse gas production in penguin guano. They showed that the penguin guano from about 1,1000 penguins emitted about 18.0 ± 10.8 kg CH_4_ in the breeding period on Fildes Peninsula. In general, previous researches on GHG emission in the Antarctic mainly focus on the site observation. There is still lack of accurate estimates of total GHG emissions from penguins in the regional or global scale.

The inaccessibility of portions of the breeding habitat for Antarctic penguins has necessitated the use of satellite imagery as a means of detecting and monitoring Adélie populations^17^. Remote sensing as an important technique has been used to monitor species abundance and distribution repeatedly, due to climate change and other environmental changes across the globe^18^. Remote sensing of penguin populations was first demonstrated with Landsat in the 1980s, and the guano at Adélie penguin colonies could be differentiated from the surrounding landscape^17,19,20^. Various remote sensing data such as Landsat, SPOT, QuickBird-2, and aerial photographs have subsequently been used to identify changes in penguin populations and their distribution^4,21–27^. Landsat-7 data was recently used to map the abundance and distribution of Adélie penguins at the continental scale^26,28^. Very High Resolution (VHR) satellite images are also a viable alternative for estimating Adélie penguin abundance and tracking changes in occupancy at a regional or continental scale^17^. For example, LaRue *et al*.^21^ used VHR images and historic aerial photographs to quantify the decadal population change of Adélie penguins on Beaufort Island in the Ross Sea. Witharana *et al*.^29^ used seven widely-used fusion algorithms to resolution enhance a series of VHR images to pave the way for more standardized products for specific types of wildlife surveys. In addition, high angle oblique aerial photographic surveys of colonies were acquired and penguins were counted during the breeding seasons in the Ross Sea during 1981–2012^4^. The Adélie penguin population was estimated for the first time in 1993 and was considered to be approximately 2.6 million breeding pairs^17^. Lynch *et al*.^26^ reported a global census of the Adélie penguin, which estimated 3.79 million breeding pairs. Many previous studies of the abundance of Antarctic penguin colonies have been conducted by considering the relationship between penguin guano areas and the number of breeding pairs^1,17,30,31^. For example, a generalized linear mixed model, with Poisson errors, was fitted to predict the abundance of breeding pairs as a function of the area of current-year guano staining identified in satellite imagery^17^. OBIA technology could greatly increase the performance of high-resolution remote sensing image classification^32^. For example, Witharana *et al*.^33^ employed geographic object-based image analysis (GEOBIA) methods to classify guano stains, indicative of chinstrap penguin and Adélie penguin breeding areas, from VHR images. However, little work directly studied penguin pixels to estimate the Adélie penguin populations using OBIA methods.

The impacts of climate change on penguins in the Antarctic are likely be highly site specific based on regional climate trends, and a southward contraction in the range of Adélie penguins is likely over the next century^3^. Some studies have shown that Adélie penguin populations have been declining rapidly on the islands of the northern Antarctic Peninsula, but have been increasing in the southern Antarctic Peninsula region and the Ross Sea region^17,34^. One previous study indicated that warming temperatures will benefit Adélie penguins, due to glacial retreat and snow melt, increases in the available habitat, and subsequent decreases in emigration rates in the southern Ross Sea region^21^. However, Adélie penguin populations are decreasing due to warming temperatures on the northern Antarctic Peninsula^35^. The driving factors of penguin populations were studied from physical changes and biological changes in the penguins’ environment. Physical environmental factors including changes in sea-ice conditions such as concentration, extent and thickness, air temperature, wind speed, sea surface temperature and so on are likely to limit the abundance of Adélie penguins^4^. Competition for food resources such as Antarctic krill (*Euphausia superba*) and continuous changes in abundance of these resources also impact the numbers and density of Adélie penguins^17,21,36,37^. In the Ross Sea sector of the Southern Ocean, Adélie penguins breed over a latitudinal range of 1,200 km^4^. The Adélie penguin has intermittently occupied the Ross Sea for ~45,000 years, and both the West Antarctic Peninsula and Ross Sea regions have been characterized by high chick-rearing habitat suitability in the recent past^3^. Inexpressible Island, on the shore of the Ross Sea, has an Adélie penguin colony. Because of the inconvenient geographic location and poor geographical circumstances, few previous studies have focused on Adélie penguin population and the impact factors on Inexpressible Island.

This study synthesized the disciplines of remote sensing, ecology, biology and soil science. The objectives of the study were: (1) to identify the number of Adélie penguins on Inexpressible Island in Victoria Land based on aerial photographs in 1983 and 2012, respectively, (2) to analyze population change and its causes from physical and biological environments, and (3) to develop a model of GHG emission estimation from marine animals, and quantitatively estimate CH_4_ and N_2_O emission potential from penguin colonies during the breeding seasons on Inexpressible Island.

## Results

### Adélie penguin population estimation based on the OBIA method

**Figure 1:**
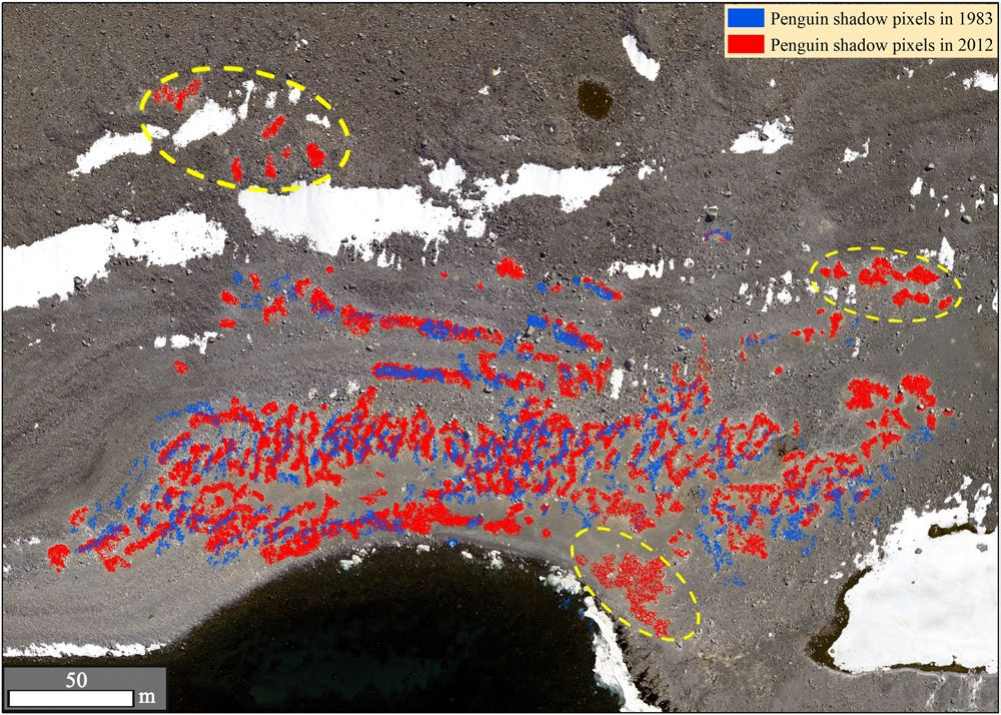
Combined map of penguin binary images in 1983 and 2012. The blue and red pixels are penguin shadow pixels in 1983 and 2012, respectively. The figure was generated by using ArcGIS 10.2 (http://www.esri.com/).

Considering low solar elevation in the Antarctic, it was assumed that the large fraction of dark areas in aerial photographs were penguin shadow pixels. The OBIA method was used to extract penguin shadow pixels directly from aerial photographs. Combining with shadow analysis, we then estimated the penguin populations in aerial photographs. Figure 1 showed that the spatial distribution of penguin shadow pixels extracted, in which the blue pixels were penguin shadow pixels in 1983 and red were in 2012. The total number of penguin shadow pixels was 236,253 in 1983 and 292,323 in 2012, respectively.

The height of an Adélie penguin is estimated to be 46–61 cm, and the chest width is 15–25 cm^38^. The average height and chest width of an Adélie penguin is set to be 53.5 and 20 cm, respectively. The shadow analysis indicated that the solar elevation was 37.72°, and the mean number of shadow pixels of one Adélie penguin in the image was 13.8. Based on the total number of penguin pixels and the shadow analysis, the total Adélie penguin population on Inexpressible Island was estimated to be 17,120 breeding pairs in 1983 and 21,183 breeding pairs in 2012, respectively. The penguin population on Inexpressible Island increased by 24% during the period from 1983 to 2012.

Compared with the penguin population in 1983, there were several new penguin colonies in 2012 (Fig. 1). For the original habitat colonies, the penguin population tended to be increasing in 2012 when compared with the results in 1983. This suggests that Adélie penguins on Inexpressible Island might be “climate change winners”.

Six samples in both gray scale image and red–green–blue (RGB) image were selected to verify the accuracy of the OBIA method. We got the value of True Positive (TP), False Positive (FP), False Negative (FN) and True Negative (TN) for each sample by analysis of intersect, erase and union. Table 1 reported the overall accuracy of OBIA method. For the gray scale image in 1983, there was only one sample whose precision was less than 70%, and the mean of *F*_*β*_ was 75.5%. For the RGB image in 2012, the Precision and Recall of six samples were more than 70%, and the mean of *F*_*β*_ was 78%. Thus overall average *F*_*β*_ was 76.8%. We can find from the table that the overall accuracy of RGB image was better than gray scale image.

**Table 1:** Accuracy assessment of object-based image analysis (OBIA) method on the estimation of penguin population.

Sample	Gray scale image			RGB image		
	Precision	Recall	*F*_*β*_	Precision	Recall	*F*_*β*_
1	0.72	0.77	0.74	0.74	0.76	0.75
2	0.79	0.81	0.80	0.89	0.75	0.81
3	0.65	0.70	0.67	0.74	0.77	0.75
4	0.77	0.78	0.77	0.85	0.72	0.78
5	0.80	0.79	0.79	0.74	0.78	0.76
6	0.76	0.77	0.76	0.84	0.82	0.83

### Estimation of total GHG emissions from penguin colonies

An estimation model on the CH_4_ and N_2_O emissions from penguin colonies was developed based on the CH_4_ and N_2_O emission factors, penguin population and the guano amount excreted during the breeding seasons. Total CH_4_ and N_2_O emissions from penguin colonies on Inexpressible Island were calculated separately in 1983 and 2012 during the penguin breeding seasons. The average dry weight of the guano produced by one penguin per day was 84.5 (g/day)^14^ and the length of the Adélie penguin breeding season was 90 days^39^. The moisture content of the penguin guano (on a dry weight basis) was determined to be 185.72%. *Z*hu *et al*.^16^ reported that the mean CH_4_ emissions from penguin guano varied from 38.22 to 219.60 μg CH_4_-C kg^−1^h^−1^ under aerobic conditions, and N_2_O varied from 0.45 to 0.74 μg N_2_O-N kg^−1^h^−1^. The averaged CH_4_ and N_2_O fluxes were 171.88 and 1.87 μg kg^−1^ h^−1^, respectively.

Table 2 showed GHG emissions and GWP of CH_4_ and N_2_O emissions from penguin colonies on Inexpressible Island in 1983 and 2012 based on average method and fitting method. It indicated that the mean relative deviation of these two methods was 5.22%, which showed that the average method was reliable if there was no enough penguin population data during the breeding season. The penguin population estimated in early December based on aerial photography might represent the average value of penguin during the breeding season in our study area. Taking the average results of two methods, total average CH_4_ and N_2_O emissions were 71.82 kg CH_4_ and 0.78 kg N_2_O in 1983, and 88.87 kg CH_4_ and 0.97 kg N_2_O in 2012, respectively. These results indicated that the total GHG emissions from Adélie penguin colonies increased significantly in 2012 when compared with that in 1983. CH_4_ was the main GHGs emitted from the penguin colonies. Total global warming potential of CH_4_ and N_2_O emissions from penguin colonies was 5302.87 kg CO_2_-eq in 1983 and 6561.43 kg CO_2_-eq in 2012, respectively.

**Table 2:** Total greenhouse gas (GHG) emissions and global warming potential (GWP) of CH_4_ and N_2_O emissions from penguin colonies on Inexpressible Island in 1983 and 2012.

Year	CH_4_ (kg)			N_2_O (kg)			GWP (kgCO_2_-eq)		
	Average method	Fitting method	Relative deviation	Average method	Fitting method	Relative deviation	Average method	Fitting method	Relative deviation
1983	69.82	73.82	5.42%	0.76	0.80	5.0%	5155.98	5449.75	5.39%
2012	86.39	91.34	5.42%	0.94	0.99	5.05%	6379.63	6743.23	5.39%

## Discussion

### Impact factors on the Adélie penguin population change

The increase of Adélie penguin population on Inexpressible Island in Ross Sea region was identified in this study, which was in agreement with the previous studies^1,21^. Lynch *et al*.^26^ found that Adélie penguin population generally increased in the Ross Sea region, while the population along the western Antarctic Peninsula had declined^1^. LaRue *et al*.^21^ reported a recent Adélie penguin population increase in response to the increased availability of nesting habitats and the receded glaciers on Beaufort Island, which was also spatially located in the Ross Sea region. In Antarctic ecosystems, environmental conditions such as air temperature, sea ice area, sea ice extent and some other environmental factors have been shown to affect the species and population dynamics^40,41^.

**Figure 2:**
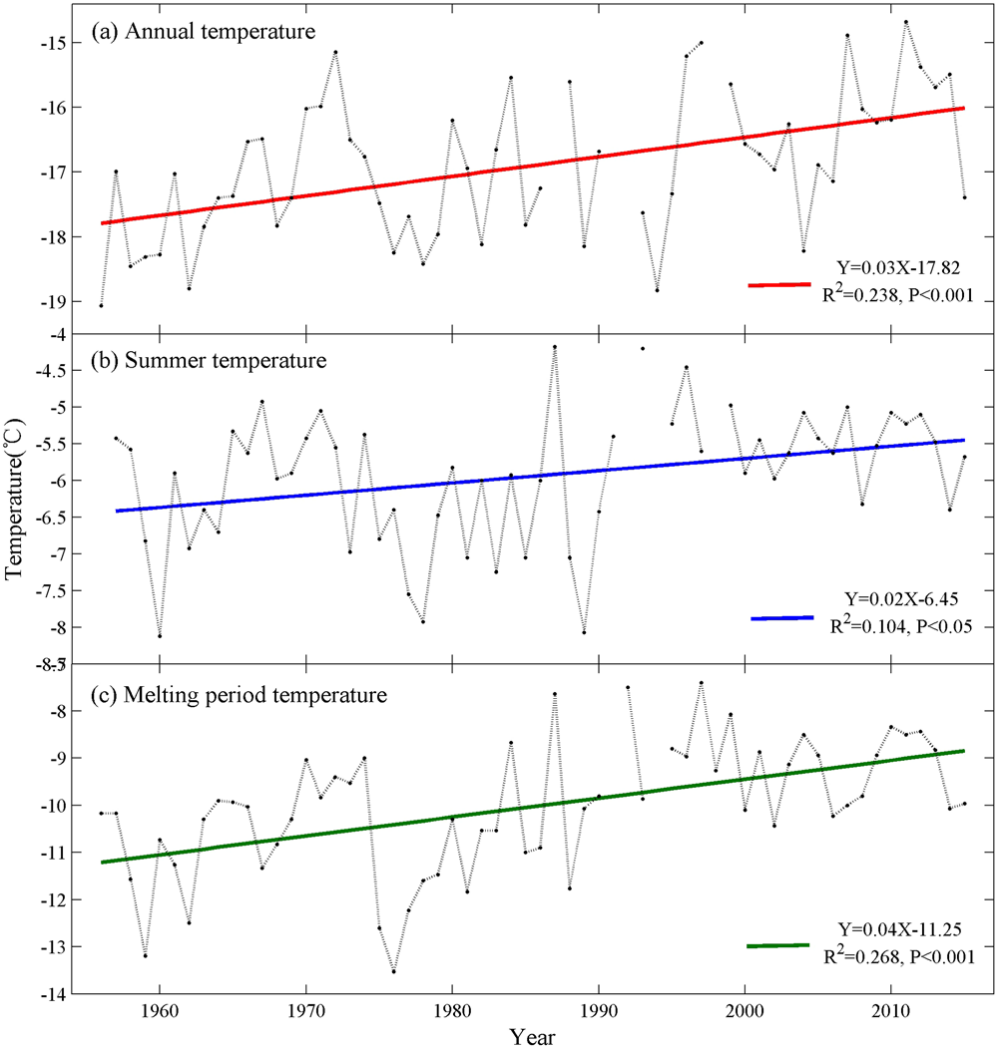
Temperature variations recorded at McMurdo station, during 1956–2015. (**a**) Average annual (January–December) temperature variations; (**b**) Average summer (December–February) temperature variations; (**c**) Average ice and snow melting period (October–December) temperature variations. The breakpoint on the time series is missing value.

In the last 60 years, the average annual temperature, summer temperature, ice and snow melting period temperature all have been increasing at McMurdo station, which was the closest meteorological station with long time serial temperature data to Inexpressible Island in this study (Fig. 2). We found that the average temperature recorded at McMurdo Station during the period of ice and snow melt within the colony had the most apparent growth trend, which increased by 2.36 °C during 1956–2015, and the average annual temperature and average summer temperature increased by 1.77 °C and 1.18 °C, respectively (Fig. 2). Figure 2 also showed that in the recent twenty years, there were less temperature fluctuation compared with the previous forty years. LaRue *et al*.^21^ found that temperatures contributed to glacial retreat and snow melt, which had increased the available habitat for Adélie penguins. In the southern Ross Sea, temperature played a role in the increases of the area of Adélie penguin nesting habitat and colony size^21^. However, in the southern Antarctic Peninsula, the warm temperatures also led to increased snowfall and decreased sea ice, with detrimental impacts on Adélie penguin colonies^21,37,42–44^. It is likely that warm temperatures influence penguin’s survival through positive and negative mechanisms according the different location of penguin colonies. We may say that the habitat release due to the increasing temperature has resulted in positive growth of Adélie penguin populations on Inexpressible Island during 1983–2012.

**Figure 3:**
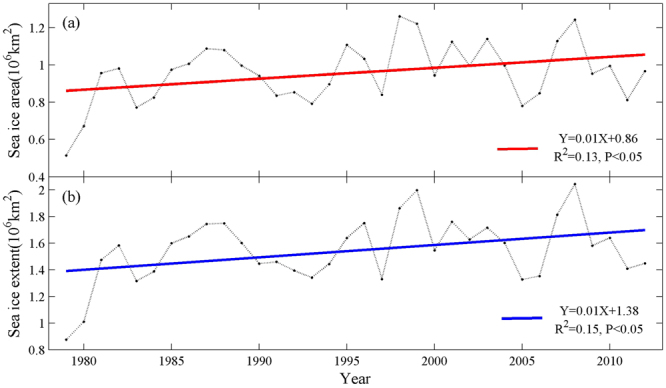
Average summer (December–February) sea ice area (**a**) and sea ice extent (**b**) variations in the Ross Sea, during 1979–2012.

The average summer sea ice area and extent in the Ross Sea increased during 1979–2012 (Fig. 3). As revealed in Fig. 3, variation tendency of the average summer sea ice area agreed with average summer sea ice extent. Average summer sea ice area and extent in the Ross Sea both increased by 0.33 million km² during 1979–2012. There were a lowest value of average summer sea ice area and extent in 1979 and two highest values in 1999 and 2008 (Fig. 3). In contrast to the western Antarctic Peninsula, the sea ice extent and duration have increased substantially over the past 40 years in the Ross Sea region^1^. The influence mechanism of sea ice on the Adélie penguin populations is quite complex. There generally exists an optimum condition in sea ice extent for Adélie penguins^41^. Too much sea ice will be not conducive to the prey of Adélie penguins because sea ice reduces access to the ocean and food^4,35,45^. While too little sea ice negatively affects the penguin resting and the abundance of Antarctic krill (*Euphausia superba*), one of the main prey items for Adélie penguins^41^. Lyver *et al*.^4^ indicated that 75% of Adélie penguin colonies in the north of 70°S would decrease or disappear by 2050 due to the disappearance of sea ice; however, the Ross Sea colonies in the south of 70°S may well be the last to benefit from the presence of sea-ice if current climate trends continue. We hypothesize that the Adélie penguin population presents a hump-shaped variation with increasing sea ice, and for now the increased sea ice has not reached the optimum value in the Ross Sea.

**Figure 4:**
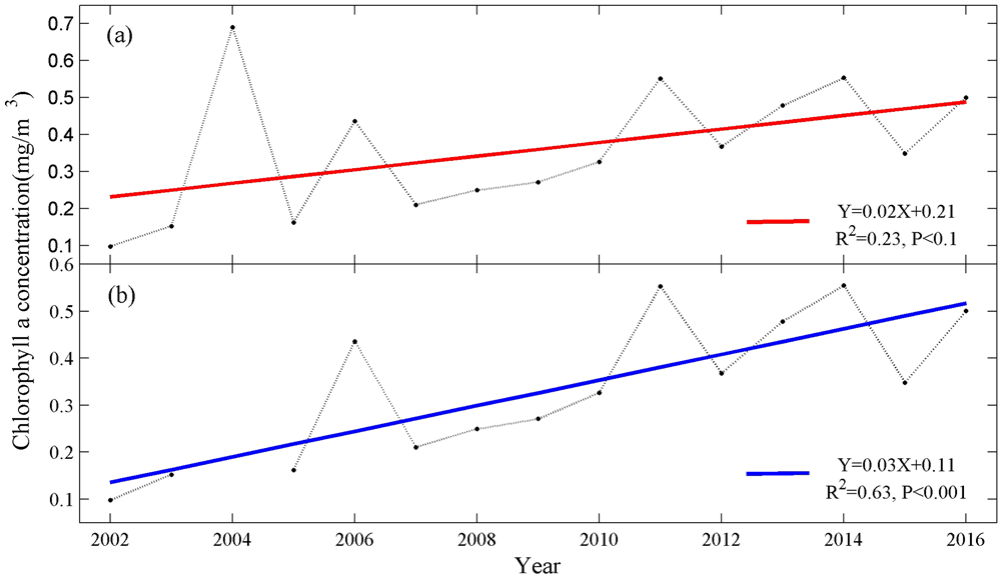
Average summer (December–February) chlorophyll-a concentration variations in the Terra Nova Bay, during 2002–2016. The figures were generated according to all data (**a**) and data without the value in 2004 (**b**).

Biological changes in the environment have also been shown to affect penguin populations. Competition for food and food availability are the most likely variables affecting the body condition of penguins and their subsequent survival or whether they return to breed in the following year^4^. Adélie penguins in the southern Ross Sea are important predators of krill and silverfish^21^. And summer phytoplankton blooms are important factors influencing Antarctic krill recruitment in the Antarctic Peninsula region, which is widely recognized as a major link between primary producers and many populations of krill-feeding vertebrates, including penguins^46–48^. The averaged summer chlorophyll-a concentration which can quantify phytoplankton blooms in the Terra Nova Bay which is adjacent to the east side of the Inexpressible Island increased during 2002–2016 (Fig. 4). The growth trend of summer chlorophyll-a concentration was much more significant when the relatively high value of 2004 was removed (Fig. 4b). The increased chlorophyll-a concentration as the indication of phytoplankton biomass will contribute to the krill availability that was the major prey food of Adélie penguin on Inexpressible Island.

### Uncertainty analysis of the GHG emission estimation

The estimation model on GHG emissions was affected by some parameters such as CH_4_ and N_2_O emission factors, penguin populations and the guano amount. The errors from all these parameters will affect the estimated uncertainty. For instance, the averaged CH_4_ and N_2_O emission factors were 171.88 μg kg^−1^ h^−1^ and 1.87 μg kg^−1^ h^−1^, respectively in this study. And all these CH_4_ and N_2_O fluxes were measured through penguin guano samples which were obtained in penguin guano profiles from penguin colonies^16^. In previous studies, Zhu *et al*.^14^ also indicated that the CH_4_ emission from 150 g fresh penguin droppings per hour was 9.0 μg h^−1^ equivalent to 60 μg kg^−1^ h^−1^. However, their CH_4_ flux was observed based on the tundra soils with fresh addition of penguin guano^14^. The different composition of penguin guano and tundra soils will lead to different potential for CH_4_ and N_2_O emissions. In the study of Zhu *et al*.^5^, the measurement results of mean CH_4_ flux was 0.108 mg CH_4_-C kg^−1^ h^−1^ equivalent to 143.33 μg CH_4_ kg^−1^ h^−1^, and the mean N_2_O flux was 0.72 μg N_2_O-N kg^−1^ h^−1^ equivalent to 2.25 μg N_2_O kg^−1^ h^−1^, which were both approximate to our study. Relatively high total organic carbon content was found in penguin guano when compared with tundra soil. The accuracy of CH_4_ and N_2_O flux factors will affect the total estimation of CH_4_ and N_2_O emissions. We found that the IPCC reported methane (CH_4_) emissions from manure management which were evaluated under different livestock production conditions. For example, the poultry in developing countries were shown to produce 30 mg CH_4_ per head per day under cool condition(10 °C < T < 15 °C)^49^. In our study, the guano of Adélie penguin produced about 1 mg CH_4_ per head per day. The discrepancy may be caused by the different computational theory and methods between IPCC and us. In addition, one study from China showed that the average annual growth rate of livestock and poultry emissions in Beijing area was 2% during the period of 1978–2009^50^. In our study, the total CH_4_ emission from penguin guano on Inexpressible Island during the penguin breeding season were 73.82 kg in 1983 and 91.34 kg in 2012, with an average annual increase by 1%. Although the results showed that total GHG emissions from one penguin were small, one study estimated the total global population of Adélie Penguins to be 3.79 million^1^. On this basis, we can estimate the total global warming potential of CH_4_ and N_2_O emissions were up to 1141 tons CO_2_-eq during the penguin breeding season.

There were several sources of estimation uncertainty about the CH_4_ and N_2_O emissions from penguin colonies on Inexpressible Island: (1) The penguin guano samples in the Zhu *et al*.^16^ we referenced were collected from Gardner Island (68°34′S, 77°52′E) and Magnetic Island (68°32′S, 77°54′E), where the climatic conditions and soil environment can be different from that on Inexpressible Island. (2) The estimations could have been influenced by the setting of parameters in the estimation model, such as the CH_4_ and N_2_O fluxes of penguin guano, the fresh weight of penguin guano, and the duration of GHG observation. For instance, in the laboratory incubation experiment referenced here, the temperature was 4 °C, but the real temperature in the field was changeable, and the penguin breeding season and amount of penguin guano may also differ from the theoretical value, all of which could lead to deviations in the estimation. (3) Our GHG emission estimation model did not account for the guano deposited by chicks and old guano in previous years on the colony. Southwell *et al*.^39^ reported that penguin eggs began to hatch in late-December and chicks became independent of the nest site in mid-January. The stay period of the chicks was short and guano deposition amount was small. The potential of GHG emissions from chicks in breeding season from mid-October to mid-January were little, which might be negligible. For the old guano from previous year, it was not presented separately in the empirical model. However, the CH_4_ and N_2_O flux factors we got from the incubation experiment in Zhu *et al*.^16^ were taken from dropping soil cores which had sedimentated for many years. To some degree, it presented the old guano emission potential together. In the future, a dynamic model including temporal and spatial information was needed to predict the CH_4_ and N_2_O emission from old and new guano deposition if we can get enough parameters in this research area. (4) The accuracy of the penguin population estimations directly affected the GHG emission estimations. Much more aerial photography and very high resolution satellite images were needed to study the population dynamics of Adélie penguin during the breeding season. Identification methodology of Adélie penguin based on remote sensing data played great role in improving the precision of penguin population.

## Materials and Methods

### Study region

**Figure 5:**
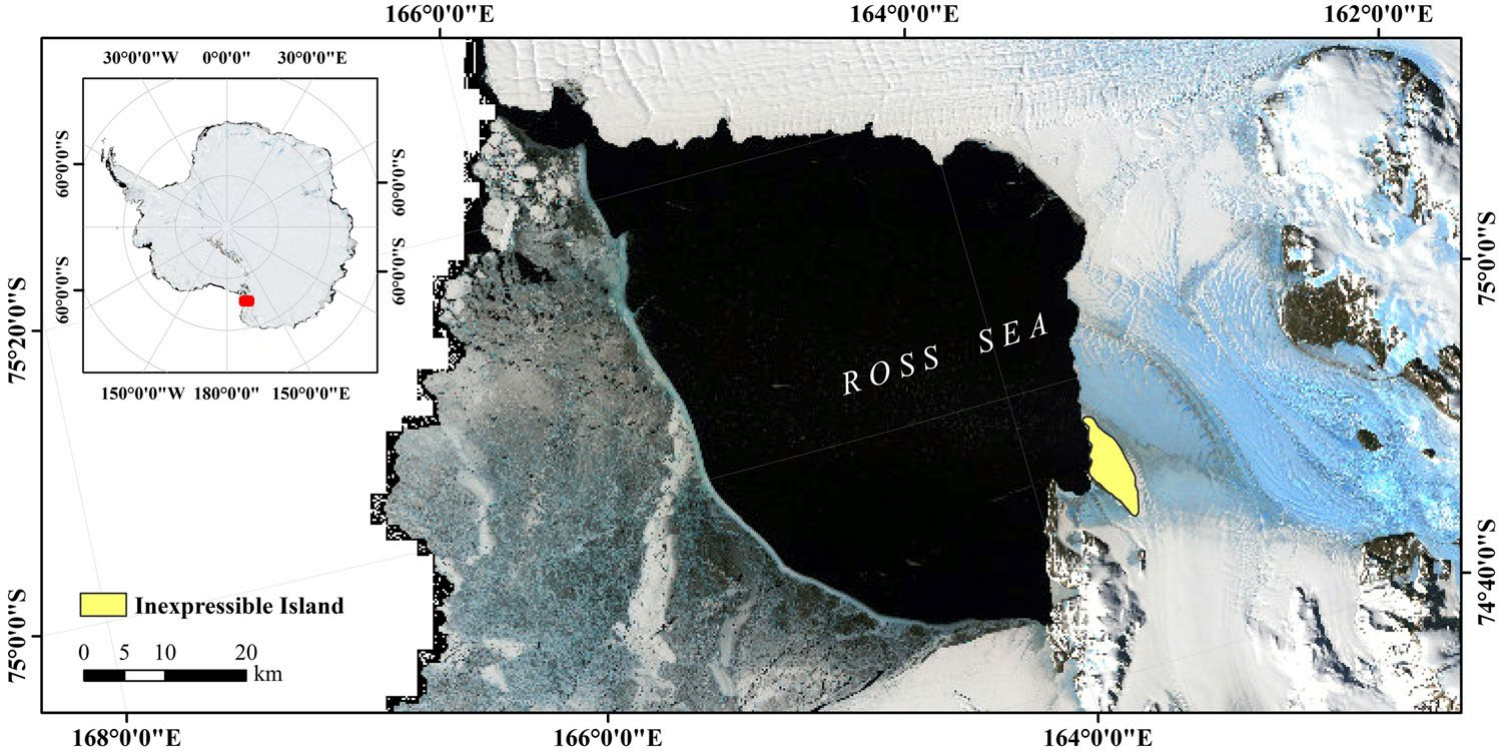
The location of Inexpressible Island in Victoria Land, Antarctic. The figure was generated by using ArcGIS 10.2 (http://www.esri.com/).

The study area was a penguin colony on Inexpressible Island (74°54′N, 163°39′E), which is a small, rocky island on the shore of the Ross Ice Shelf in Terra Nova Bay, Victoria Land, Antarctica, with an area of 70 km^2^ (Fig. 5). The Northern Party, a British expedition, surveyed here in 1910–1913, and named the island, the Southern Foothills and the Northern Foothills. During the survey, the team spent the winter in miserable conditions because of a shortage of supplies, and therefore the island was renamed “Inexpressible Island”. The island faces the Ross Sea and contains snow-covered mountains. There is strong wind on the island throughout the year, which can reach in excess of level 8 on the Beaufort scale. The ice and snow melt quickly on the island in summer, leaving only the shore and mountains covered with snow and ice. The 31^st^ Chinese Antarctica research expedition conducted a detailed geological exploration of the island, and found eight kinds of lichen. There are many gravels produced by the glacier dynamics, among which there is a penguin colony, with tens of thousands of penguins breeding every summer.

### Data sources

**Figure 6:**
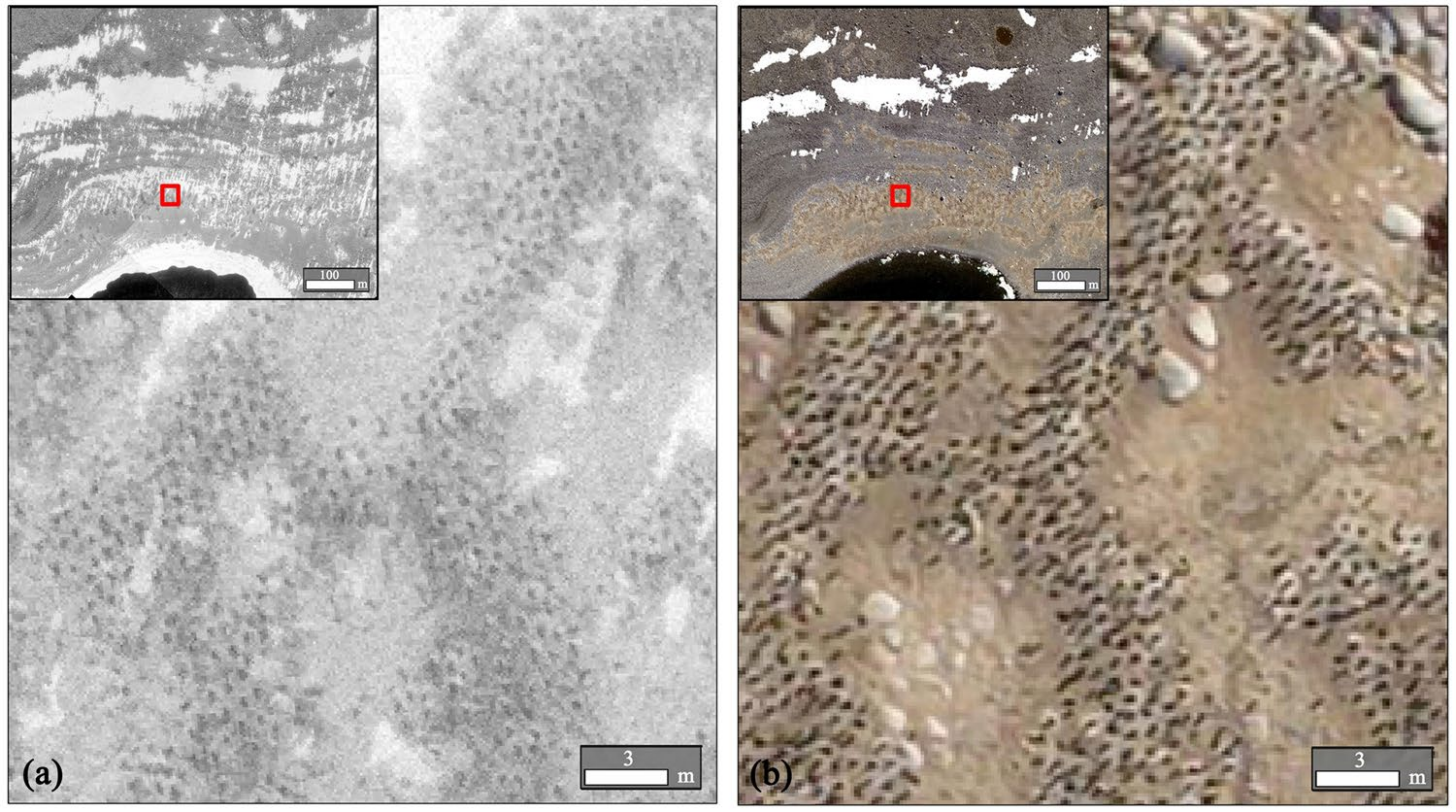
Aerial photographs of penguin colonies in Victoria Land, Antarctic, in 1983 (**a**) and 2012 (**b**). The figure was generated by using ArcGIS 10.2 (http://www.esri.com/).

We used aerial photographs of Inexpressible Island that were taken in early December in 1983 and 2012. The colony population was represented almost entirely by one member of each penguin pair incubating its eggs, and minimal numbers of non-breeders not on territories^21,51^. The photographs in 1983 were gray scale image (Fig. 6a), and in 2012 were RGB image (Fig. 6b). The dark areas in the images were penguin shadow pixels and can be clearly distinguished (Fig. 6). Table 3 showed characteristics of two data products.

**Table 3:** Characteristics of the gray scale image in 1983 and red-green-blue image in 2012.

Year	Image type	Resolution(m)	Source	Shooting height(m)	Camera type
1983	Gray scale image	0.1–0.38	The University of Minnesota	800	WILDRC8
2012	Red–Green–Blue (RGB) image	0.1	The 29th Chinese Antarctica research expedition	500	Hasselblad H4D60

The available temperature records were gathered from McMurdo Station (http://www.antarctica.ac.uk/met/READER/), which is located on Ross Island. McMurdo Station is the closest station to the study area with a long-term data series. In fact, the Mario Zucchelli Station is the nearest station to Inexpressible Island, but there is no long time serial temperature data. Sea ice area and extent data in the Ross Sea were obtained from NASA (http://neptune.gsfc.nasa.gov). The ocean chlorophyll-a concentration data used to quantify phytoplankton blooms were obtained from the Ocean Biology Processing Group, Global Change Data Center, Earth Sciences Division, Science and Exploration Directorate, Goddard Space Flight Center, NASA (http://oceancolor.gsfc.nasa.gov), with a spatial resolution of 4 km^2^.

### Estimation of the Adélie penguin population based on the OBIA method

Original gray scale images in 1983 and RGB images in 2012 were georeferenced by seven tie points, such as cliff peaks and boulders, in the penguin colonies using the ArcGIS 10.2 (ESRI Inc., USA). The tie-points we selected were distributed as evenly as possible, and total RMSR was 3.02. The penguin dense regions, as shown by dark pixels, were manually interpreted. The OBIA method was used to extract the penguin shadow pixels from the penguin dense regions with three steps, namely segmentation, classification, and artificial modification, respectively. All these steps were implemented on Easy Interpretation (Beijing reavenue technology company, China) software package. The segmentation algorithm we used was multiresolution. Optimal segmentation results derived from the optimal segmentation scale, and left other parameters such as smoothness by default. In this study, optimal scale of 15 was determined empirically through try-and-error experiments. In the second step, we used rule-based classification method to extract penguin shadow pixels. Specifically, the attributes we adopted to construct classification rule were brightness index and mean deviation index. We chose some penguin objects and other objects to compare the difference in these two indexes, based on which the rule expressions of classification were determined. The final criteria of penguin objects were that the brightness index was more than 0, and the mean deviation index was less than 0.85. After the segmentation and classification, in order to improve the accuracy of the extraction results, we used a rule-based approach refine the previous classification. Specifically, we thought that objects extracted as penguin shadow larger than 45 pixels should be manual checked. The threshold determination of area mainly depended on expert knowledge and multiple experiments.

To calculate the total number of penguin shadow pixels, penguin polygons exported from Easy Interpretation software package were converted to penguin binary images. The shadow analysis set up a bridge between penguin shadow pixels and penguin numbers. The shadow analysis model had two components: (1) the calculation of the solar elevation, and (2) the mean number of shadow pixels of one Adélie penguin in the image. For the first component, the solar zenith angle were first calculated according to the local latitude, solar declination, and solar hour angle (Eqns 1 and 2), and then the solar elevation was obtained based on the relationship of the solar zenith angle and solar elevation (Eqn. 3). For the other components, the mean number of shadow pixels of one Adélie penguin was a function of the solar elevation, the average size of penguins (e.g., height and chest width), and the resolution of the aerial photograph. Then, the number of Adélie penguins could be calculated by the total number of penguin shadow pixels divided by the mean number of shadow pixels of one Adélie penguin in the image.

1 $$\cos \,z=\,\sin \,{\theta }\,\sin \,{\delta }+\,\cos \,{\theta }\,\cos \,{\delta }\,\cos \,{\omega }$$

2 $${\omega }={l}_{d}-l$$

3 $$\alpha =90-Z$$

4 $$p=(H/\tan \,\alpha )\times W/{R}^{2}$$

where α is solar elevation; *Z* is solar zenith angle; *θ* is local latitude; *δ* is solar declination, which can be found in the table of solar declinations (http://www.starpath.com); *ω* is solar hour angle; *l*_*d*_ is the longitude of the subsolar point; *l* is local longitude; *p* is the average number of penguin shadow pixels of one penguin, *H* is the average height of an Adélie penguin; *W* is the average chest width of an Adélie penguin, and *R* is the resolution of the aerial photograph.

To verify the accuracy of the OBIA method, six penguin colony samples were selected from each of the color image in 2012 and gray scale image in 1983. The OBIA method and visual interpretation were used to estimate the penguin populations of each sample, respectively. It was supposed that pixels were divided into four types: TP, FP, FN, and TN. TP pixel was extracted as penguin pixel, and was actually penguin pixel. FP pixel was extracted as penguin pixel, and was not actually penguin pixel. FN pixel was not extracted as penguin pixel, and was actually penguin pixel. TN pixel was not extracted as penguin pixel, and was not actually penguin pixel. Then we used the index of precision, recall and *F*_*β*_ to verify the accuracy of the OBIA method^33^. In ArcGIS 10.2 (ESRI Inc., USA), penguin population results of each sample were analyzed by the analysis tools such as intersect, erase and union to get the value of TP, FP, FN and TN.

5 $${\rm{Precision}}=\frac{TP}{TP+FP}$$

6 $${\rm{Re}}\,call=\frac{TP}{TP+FN}$$

7 $${F}_{\beta }=\frac{(1+{\beta }^{2})\times {\rm{\Pr }}\,ecision\times {\rm{Re}}\,call}{{\beta }^{2}\times {\rm{\Pr }}\,ecision+{\rm{Re}}\,call}$$

Precision indicates the fraction of pixels extracted as positive that are correct. Recall indicates the fraction of penguin pixels that are extracted. *F*_*β*_ is the weighted average of precision and recall. Because both the precision and recall in our study were equally important, we defined the value of *β* as 1.

The change of Adélie penguin population on Inexpressible Island from 1983 to 2012 was studied, and impact factors on the change were considered in relation to the physical and biological environments. To study the influence of temperature on penguin populations, the average annual (January–December) temperature, summer (December–February) temperature, ice and snow melting period (October–December) temperature at McMurdo station during 1956–2015 were calculated. And the average summer (December–February) sea ice area and extent in the Ross Sea region during 1979–2012 were calculated to study the sea ice variation. The phytoplankton blooms variation was studied through the ocean chlorophyll-a concentration data. The chlorophyll-a concentration data we download were global data. A mask was used to extract the chlorophyll-a concentration data in the Terra Nova Bay which is adjacent to the east side of the Inexpressible Island. Then the average summer (December–February) chlorophyll-a concentration in the Terra Nova Bay during 2002–2016 were calculated.

### Estimation of the total GHG emissions from penguin colonies

GHG fluxes can be acquired by field observation and laboratory simulation. The net CH_4_ and N_2_O fluxes were usually determined by a static chamber technique^52,53^. Based on simulation experiments under laboratory conditions, Sun *et al*. and Zhu *et al*. have done some research about GHG fluxes^5,10,12,14,16^. Zhu *et al*.^16^ collected Adélie penguin guano samples during the 22nd Chinese Antarctica research expedition. They incubated penguin guano in the lab setting and collected CH_4_ and N_2_O gas samples frequently. Zhu *et al*.^16^ reported that the mean CH_4_ emissions from penguin guano varied from 38.22–219.60 μg CH_4_-C kg^−1^h^−1^ under aerobic conditions in lab settings. The average of maximum and minimum CH_4_ fluxes (μg CH_4_-C kg^−1^ h^−1^) which was converted to unit of μg CH_4_ kg^−1^ h^−1^ was used to present the CH_4_ production potential, as well as the N_2_O. The averaged CH_4_ and N_2_O fluxes were 171.88 and 1.87 μg kg^−1^ h^−1^, respectively. These values showed the CH_4_ and N_2_O production potentials in penguin guano and we used these values to calculate the total CH_4_ and N_2_O emission potentials based on penguin population, breeding season, fresh weight of guano and so on. The model described in Eqns (8) and (9) was built to estimate GHG emission potential from penguin colonies. The fresh guano produced every day was calculated based on the dry weight and moisture content (on a dry weight basis). The global warming potential (GWP) of CH_4_ and N_2_O were 25 and 298 times higher than that of CO_2_ on 100-year horizon (Eqn. (10)).

8 $${F}_{(C{H}_{4}/{N}_{2}O)}=\sum _{1}^{t}t\times {N}_{t}\times G\times {10}^{-3}\times {f}_{(C{H}_{4}/{N}_{2}O)}\times 24\times {10}^{-9}$$

9 $$G={M}_{C}\times D+D$$

10 $$GWP=\frac{{F}_{C{H}_{4}}}{16}\times 44\times 25+\frac{{F}_{{N}_{2}O-N}}{28}\times 44\times 298$$

where *t* denotes the all breeding season days with the values from 1 to 90, *N*_*t*_ is the number of Adélie penguin breeding pairs in different stage of breeding season, *G* is the fresh weight of guano produced by one penguin per day (g), *D* is the average dry weight of guano produced by one penguin per day (g), *f*_*(CH4/N2O)*_ is the emission flux (μg kg^−1^ h^−1^) of different greenhouse gases, and *M*_*c*_ is the moisture content of the penguin guano (on a dry weight basis).

In this study, the number of Adélie penguin we got from aerial photography data was estimated in December when only one member of each penguin pair remained in the colony in most circumstances. It is difficult to detect the changes of actual penguin population due to a lack of continuous observation data. Therefore, we adopt two methods named average method and fitting method to represent penguin population in the breeding season. For the average method, we used the number of Adélie penguin we got from aerial photography data as the average population. For the fitting method, we tried to simulate the dynamic change of penguin population in the breeding season based on the previous studies^39,54^, and we divided the breeding season into three stages as follows to estimate the variation of penguin population. The first stage is from mid-October to mid-November (about 30 days) when Adélie penguins start arriving at their land-based breeding colonies, the penguin population varies from 0 to peak value which is approximately twice of the population in December calculated in our study. The second stage is from mid-November to late-November (about 15 days) when females leave to forage, the penguin population varies from peak value to the population in December. The third stage is from late-November to mid-January (about 45 days) when females and males leave the breeding sites in turn to forage and the number of attending adults generally remains constant. We hypothesized that there is linear relationship between the penguin population and breeding time. We performed linear interpolation method to estimate the variations of penguin population in these three stages. The results were as follows:

11 $$\begin{array}{l}{N}_{1}=\frac{{N}_{0}}{15}\times t\quad \quad \quad \quad \quad \quad \,(0 < {\rm{t}}\le \mathrm{30})\\ {N}_{2}=-\frac{{N}_{0}}{15}\times t+4\times {N}_{0}\quad (30 < {\rm{t}}\le 45)\\ {N}_{3}={N}_{0}\quad \quad \quad \quad \quad \quad \quad \quad \,(45 < {\rm{t}}\le 90)\end{array}\}$$

where *N*_1_, *N*_2_, and *N*_3_ are the number of Adélie penguins on the colony in different stage of breeding season, respectively, *N*_0_ is the number of Adélie penguin we got from aerial photography data, *t* denotes the all breeding season days with the values from 1 to 90.

We compared the differences of CH_4_ emission, N_2_O emission, and GWP of CH_4_ and N_2_O based on average method and fitting method. Relative deviation was used to evaluate the differences between these two methods.

## References

[1] Lynch HJ, Larue MA (2015). First global census of the Adélie Penguin. Auk.

[2] Taylor RH, Wilson PR (1990). Recent increase and southern expansion of Adélie Penguin populations in the Ross Sea, Antarctica, related to climatic warming. New Zealand Journal of Ecology.

[3] Cimino MA, Lynch HJ, Saba VS, Oliver MJ (2016). Projected asymmetric response of Adélie penguins to Antarctic climate change. Scientific Reports.

[4] Lyver POB (2014). Trends in the Breeding Population of Adélie Penguins in the Ross Sea, 1981–2012: A Coincidence of Climate and Resource Extraction Effects. Plos One.

[5] Zhu R (2009). Greenhouse gas emissions from penguin guanos and ornithogenic soils in coastal Antarctica: Effects of freezing–thawing cycles. Atmospheric Environment.

[6] Tatur A, Myrcha A, Niegodzisz J (1997). Formation of abandoned penguin rookery ecosystems in the maritime Antarctic. Polar Biology.

[7] Sun L (2004). A geochemical method for the reconstruction of the occupation history of a penguin colony in the maritime Antarctic. Polar Biology.

[8] Sun L, Zhu R, Xie Z, Xing G (2002). Emissions of nitrous oxide and methane from Antarctic tundra: role of penguin dropping deposition. Atmospheric Environment.

[9] Zhu R (2006). *Tropospheric Phosphine and Its Source*s in Coastal Antarctica. Environmental Science & Technology.

[10] Zhu R, Liu Y, Xu H, Ma D, Jiang S (2013). Marine animals significantly increase tundra N2O and CH4 emissions in maritime Antarctica. Journal of Geophysical Research Biogeosciences.

[11] Zhu R (2008). Methane emissions from three sea animal colonies in the maritime Antarctic. Atmospheric Environment.

[12] Zhu, R. *et al*. Nitrous oxide emissions from sea animal colonies in the maritime Antarctic. *Geophysical Research Letters* **35**, 10.1029/2007GL032541 (2008).

[13] Zhu R, Liu Y, Sun L, Xu H (2007). Methane emissions from two tundra wetlands in eastern Antarctica. Atmospheric Environment.

[14] Zhu R, Sun L (2005). Methane fluxes from tundra soils and snowpack in the maritime Antarctic. Chemosphere.

[15] Gregorich EG (2006). *Emission of CO2, CH4 and N2O f*rom lakeshore soils in an Antarctic dry valley. Soil Biology & Biochemistry.

[16] Zhu RB (2009). Nutrient compositions and potential greenhouse gas production in penguin guano, ornithogenic soils and seal colony soils in coastal Antarctica. Antarctic Science.

[17] Larue MA (2014). A method for estimating colony sizes of Adélie penguins using remote sensing imagery. Polar Biology.

[18] Horning, N., Robinson, J. A. & Sterling, E. J. *Remote sensing for ecology and conservation: a handbook of techniques*. (Oxford University Press, 2010).

[19] Schwaller MR, Benninghoff WS, Olson CE (1984). Prospects for satellite remote sensing of Adélie penguin rookeries. International Journal of Remote Sensing.

[20] Schwaller MR, Olson CE, Ma Z, Zhu Z, Dahmer P (1989). A remote sensing analysis of Adélie penguin rookeries. Remote sensing of environment.

[21] Larue MA (2013). Climate change winners: receding ice fields facilitate colony expansion and altered dynamics in an Adélie penguin metapopulation. Plos One.

[22] Bhikharidas AK, Whitehead MD, Peterson JA (1992). Mapping Adélie penguin rookeries in the Vestfold Hills and Rauer Islands, east Antarctica, using SPOT HRV data. International Journal of Remote Sensing.

[23] Chamaillé-Jammes S, Guinet C, Nicoleau F, Argentier M (2000). A method to assess population changes in king penguins: the use of a Geographical Information System to estimate area-population relationships. Polar Biology.

[24] Fretwell PT, Trathan PN (2009). Penguins from space: faecal stains reveal the location of emperor penguin colonies. Global Ecology and Biogeography.

[25] Naveen R, Lynch HJ, Forrest S, Mueller T, Polito M (2012). First direct, site-wide penguin survey at Deception Island, Antarctica, suggests significant declines in breeding chinstrap penguins. Polar Biology.

[26] Lynch HJ, Schwaller MR (2014). Mapping the Abundance and Distribution of Adélie Penguins Using Landsat-7: First Steps towards an Integrated Multi-Sensor Pipeline for Tracking Populations at the Continental Scale. Plos One.

[27] Fretwell PT (2012). An emperor penguin population estimate: the first global, synoptic survey of a species from space. PLoS One.

[28] Schwaller MR, Southwell CJ, Emmerson LM (2013). Continental-scale mapping of Adélie penguin colonies from Landsat imagery. Remote Sensing of Environment.

[29] Witharana C, Larue MA, Lynch HJ (2016). Benchmarking of data fusion algorithms in support of earth observation based Antarctic wildlife monitoring. Isprs Journal of Photogrammetry & Remote Sensing.

[30] Fretwell PT (2012). Correction: An Emperor Penguin Population Estimate: The First Global, Synoptic Survey of a Species from Space. Plos One.

[31] Woehler EJ, Riddle MJ (1998). Spatial relationships of Adélie penguin colonies: implications for assessing population changes from remote imagery. Antarctic Science.

[32] Wei, W., Chen, X. & Ma, A. Object-oriented information extraction and application in high-resolution remote sensing image. Proceedings of the *IGARSS* 3803–3806 (2005).

[33] Witharana C, Lynch H (2016). An Object-Based Image Analysis Approach for Detecting Penguin Guano in very High Spatial Resolution Satellite Images. Remote Sensing.

[34] Lynch HJ, Naveen R, Trathan PN, Fagan WF (2012). Spatially integrated assessment reveals widespread changes in penguin populations on the Antarctic Peninsula. Ecology.

[35] Ainley D (2010). Antarctic penguin response to habitat change as Earth’s troposphere reaches 2 °C above preindustrial levels. Ecological Monographs.

[36] Bricher, P. K., Lucieer, A. & Woehler, E. J. *Population trends of Adélie penguin (Pygoscelis adeliae) breeding colonies: a spatial analysis of the effects of snow accumulation and human activities*. (Matson Museum of Anthropology, Pennsylvania State University, 2008).

[37] Fraser, W. & Patterson, D. Human dis*turba*nce and long-term changes in Adlie penguin populations: a natural experiment at Palmer Station, Antarctica. *Bataglia, B., valencia, J. & walton, D. W. H.* (1997).

[38] Williams, T. D. *Penguins*. (British Antarctic Survey, 1991).

[39] Southwell C, McKinlay J, Emmerson L, Trebilco R, Newbery K (2010). Improving estimates of Adélie penguin breeding population size: developing factors to adjust one-off population counts for availability bias. CCAMLR Science.

[40] Croxall JP, Trathan PN, Murphy EJ (2002). Environmental change and Antarctic seabird populations. Science.

[41] Jenouvrier S, Barbraud C, Weimerskirch H (2006). Sea ice affects the population dynamics of Adélie penguins in Terre Adélie. Polar Biology.

[42] Ducklow HW (2007). Marine pelagic ecosystems: the west Antarctic Peninsula. Philosophical Transactions of the Royal Society B Biological Sciences.

[43] Turner J, Overland JE, Walsh JE (2007). An Arctic and antarctic perspective on recent climate change. International Journal of Climatology.

[44] Trivelpiece WZ (2011). From the Cover: Variability in krill biomass links harvesting and climate warming to penguin population changes in Antarctica. Proceedings of the National Academy of Sciences of the United States of America.

[45] Smith RC (1999). Marine Ecosystem Sensitivity to Climate Change Historical observations and paleoecological records reveal ecological transitions in the Antarctic Peninsula region. BioScience.

[46] Casanovas P, Naveen R, Forrest S, Poncet J, Lynch HJ (2015). A comprehensive coastal seabird survey maps out the front lines of ecological change on the western Antarctic Peninsula. Polar biology.

[47] Marrari M, Daly KL, Hu C (2008). Spatial and temporal variability of SeaWiFS chlorophyll a distributions west of the Antarctic Peninsula: Implications for krill production. Deep Sea Research Part II: Topical Studies in Oceanography.

[48] Ballerini T (2014). Productivity and linkages of the food web of the southern region of the western Antarctic Peninsula continental shelf. Progress in Oceanography.

[49] (IPCC), I. P. o. C. C. *Guidelines for National Greenhouse Gas Inventories*. (Institute for Global Environmental Strategies, 2006).

[50] Liu Y, Liu J, Wu W (2013). Spatiotemporal dynamics of greenhouse gases emissions from livestock and poultry in Beijing area during 1978-2009. Chinese Journal of Eco-Agriculture.

[51] Ainley, D. *The Ad lie Penguin: Bellwether of Climate Change*. (Columbia University Press, 2002).

[52] Bartlett KB, Crill PM, Sass RL, Harriss RC, Dise NB (1992). Methane emissions from tundra environments in the Yukon-Kuskokwin Delta, Alaska. Journal of Geophysical Research Atmospheres.

[53] Mosier AR, Delgado JA, Cochran VL, Valentine DW, Parton WJ (1997). Impact of agriculture on soil consumption of atmospheric CH 4 and a comparison of CH 4 and N 2 O flux in subarctic, temperate and tropical grasslands. Nutrient Cycling in Agroecosystems.

[54] Southwell C (2015). Re-constructing historical Adélie penguin abundance estimates by retrospectively accounting for detection bias. PloS one.

